# Following the genes: a framework for animal modeling of psychiatric disorders

**DOI:** 10.1186/1741-7007-9-76

**Published:** 2011-11-11

**Authors:** Kevin J Mitchell, Z Josh Huang, Bita Moghaddam, Akira Sawa

**Affiliations:** 1 Smurfit Institute of Genetics and Institute of Neuroscience, Trinity College Dublin, Dublin 2, Ireland; 2 Cold Spring Harbor Laboratory, Cold Spring Harbor, NY 11724, USA; 3 Department of Neuroscience, University of Pittsburgh, Pittsburgh, PA 15260, USA; 4 Department of Psychiatry and Behavioral Sciences and Department of Neuroscience, Johns Hopkins University School of Medicine, Baltimore, MD 21287, USA

**Keywords:** autism, schizophrenia, rare mutations, synaptic, interneurons, EEG, functional connectivity, microcircuits, Cre, allelic heterogeneity

## Abstract

The number of individual cases of psychiatric disorders that can be ascribed to identified, rare, single mutations is increasing with great rapidity. Such mutations can be recapitulated in mice to generate animal models with direct etiological validity. Defining the underlying pathogenic mechanisms will require an experimental and theoretical framework to make the links from mutation to altered behavior in an animal or psychopathology in a human. Here, we discuss key elements of such a framework, including cell type-based phenotyping, developmental trajectories, linking circuit properties at micro and macro scales and definition of neurobiological phenotypes that are directly translatable to humans.

## Historical approaches to modeling psychiatric disorders

Psychiatric disorders constitute a diverse set of conditions, variously impinging on all domains of mental function and affecting the most fundamental human attributes: language, thought, perception, mood and sense of self. Collectively, they cause a substantial public health burden, greater than cancers or cardiovascular disease [1]. The proportional burden is actually increasing as we learn more about the molecular bases of other common diseases and how to treat them, through the application of molecular genetics. Until recently, these approaches have been difficult in psychiatry as we have had few entry points to the underlying molecular mechanisms. This is now changing as psychiatric genetics reveals more and more specific mutations predisposing to psychiatric disease. These discoveries, along with revolutionary advances in neurogenetic techniques, provide the means to elucidate pathogenic mechanisms through modeling such mutations in animals and defining their effects at the neurobiological level.

In the absence of such information on specific genetic causes, previous modeling approaches for psychiatric disorders have been based either on surface similarities between behavioral assays in rodents and domains of psychopathology in humans or on dissecting the psychopharmacological mechanisms of known drugs [2,3]. These have both centered on the goal of generating relevant behavioral assays that can provide platforms for drug screening.

The behavioral approach first defines behavioral assays in animals that are thought to mimic symptoms in particular psychological domains [4,5]. Thus, 'learned helplessness' is thought to model depressive symptoms, spontaneous alternation or latent inhibition are believed to index working memory, which is often deficient in schizophrenia, and decreased social interactions in rodents are thought to relate to similar effects in humans with autism. Animals can be generated with defects in any of these or similar domains by a variety of manipulations, including pharmacological, surgical, experiential and genetic. The relevance (or predictive validity) of these models to human psychopathology has generally been tested by whether they are responsive to known medications, such as antidepressants or antipsychotics [3]. Using these approaches, it has been possible to generate a large number of such models, which have been used to screen through drug libraries for novel therapeutic compounds or to test prototypes of existing medication. Unfortunately, these approaches have yielded few new drugs [6,7].

There are several drawbacks to using only the criterion of response to known medication. First, most of the models do not have causal etiological validity - acute blockade of NMDA-type receptors for glutamate or ventral hippocampal lesion are not actual causes of schizophrenia, for example. Second, the surface parallels between rodent and human behavior may be misleading. On the contrary, we should expect some of the underlying neurobiological defects to manifest at the behavioral level in species-specific ways, which may or may not appear related [2]. Third, defining predictive validity based on responsiveness to known drugs inevitably restricts the focus to mechanisms affected by those drugs. This circularity has undoubtedly contributed to the paucity of new drug mechanisms being found.

A complementary approach is now becoming possible as particular mutations causing psychiatric illness are being identified at an ever-increasing rate. Mouse models of these mutations can now be investigated using increasingly powerful and sophisticated tools to get at the underlying neurobiology and elucidate pathogenic mechanisms, from the level of synapses formed between specific cell types in specific circuits to the level of dynamic function of neuronal networks and concomitant moment-to-moment behavior. These types of approaches should provide the necessary neurobiological knowledge to enable the rational design of new therapeutics.

## A revolution in psychiatric genetics

The field of psychiatric genetics is undergoing a profound paradigm shift. For several decades, the prevailing model has held that psychiatric disorders arise in any individual due to the cumulative effects of a large number of common variants [8-10]. Each of these, by themselves, would have a very small effect on risk, but when the collective burden of such alleles passes a putative threshold, the system would be pushed into a pathogenic state. Though this model has little empirical support, it provided the theoretical foundation for genome-wide association studies (GWAS) [11], which aim to detect such variants by comparing allele frequencies for millions of such variants between cases and controls [12-15]. For psychiatric disorders, these studies, now carried out on tens of thousands of people, have yielded a number of replicated common variants reaching the threshold for genome-wide significance [16-26]. These include eight loci for schizophrenia [26,27], two for bipolar disorder [25] and one for schizophrenia and bipolar disorder together [25]; three potential loci arising from individual GWAS for autism [22-24] have not yet been replicated on the same scale. These findings point to loci that may be involved in disease risk at a population level but do not identify or speak to the likely allelic frequency of causal variants [28]. Each of the associated variants has a tiny statistical effect on risk at the population level and, collectively, the significant single nucleotide polymorphisms statistically account for only a few percent of the overall heritability of the disorder [18,25,26].

The alternative model is that psychiatric disorders arise due to mutations in any of a very large number of genes [11,29-33]. Under this model, psychiatric diagnostic categories are actually umbrella terms for large numbers of distinct genetic disorders that happen to result in similar spectra of symptoms. This is the sort of genetic heterogeneity that underlies categories such as congenital deafness, epilepsy, mental retardation, retinitis pigmentosa, many cancers and other conditions [34,35].

It has, of course, been known for some time that psychiatric illness could arise due to single mutations. Well-known examples include fragile × syndrome [36], Rett syndrome [37] and mutations in the *neuroligin *genes *NLGN3 *and *NLGN4 *[38], all of which are associated with autistic spectrum disorder, and velocardiofacial syndrome (22q11.2 deletion syndrome) [39] and the Scottish DISC1 translocation [40], which are associated with schizophrenia and other psychiatric diagnoses. The number of such identified mutations is now steadily and rapidly increasing, thanks to the application of new genomic microarray [41,42] and sequencing technologies [43,44], to the point where they collectively explain an appreciable fraction of psychiatric diagnoses.

Copy number variants (deletions or duplication of chromosomal segments, often affecting multiple genes) have been most readily identified and make up an important class of causal mutations in schizophrenia [42,45-51], autism [41,52-54], attention deficit-hyperactivity disorder [55-58], Tourette syndrome [59], developmental delay and mental retardation [60,61], epilepsy [62] and cortical malformations [63]. Whole-exome and whole-genome sequencing approaches are now also identifying large numbers of point mutations individually responsible for psychiatric conditions [64-71].

A number of important principles have emerged from these studies. First, there is considerable overlap in the genetic etiology of what had previously been considered distinct disorders. Individual mutations that predispose to one class of psychiatric illness, such as schizophrenia, are also associated with other disorders, such as bipolar disorder, autism, mental retardation, epilepsy, attention deficit hyperactivity disorder and Tourette's syndrome (for example, [31,72-77]), in agreement with recent epidemiological data indicating shared risk [78-83]. Traditional diagnostic categories, although still very useful in organizing daily practice in psychiatry, may therefore represent not natural kinds in terms of etiology, but more or less distinct phenotypic endpoints that may arise from common origins.

Second, the mutations so far discovered are characterized by incomplete penetrance and variable expressivity [31,77,84]. As with the DISC1 translocation, such mutations may result in a range of phenotypes and many carriers may be unaffected by any psychiatric condition [85]. Of course, the penetrance depends on which phenotype is being assessed - it will be lowest for specific diagnoses, higher for psychiatric illness generally and higher still for neurobiological endophenotypes, which may be apparent even in clinically unaffected carriers.

Third, many of the identified genes are involved in neural development [42,77,86]. While certainly not exclusive, this is probably the largest category of susceptibility genes. Genes involved in activity-dependent synaptic plasticity, such as *FMR1*, are also highly represented. With increasing numbers of genes being identified all the time, it is becoming possible to assign many of them to specific biochemical pathways or cellular processes, such as synapse formation and plasticity (for example, [64,66,87-89]).

Fourth, allelic specificity and dosage are extremely important. Different mutations in the same gene may result in very different phenotypic outcomes. As a classic example, Duchenne muscular dystrophy and Becker's muscular dystrophy are caused by different types of mutations in the *dystrophin *gene: their clinical severity, manifestation, and age of onset are also different [90]. Similar effects are seen for genes implicated in neurological and psychiatric disorders [32,35], as described later. In addition, some alleles may cause severe neurological disorders when homozygous but manifest as psychiatric illness in heterozygous form [32,35,91].

Fifth, while these findings highlight the importance of rare single mutations, they do not necessarily imply a simple mode of inheritance. Many of the mutations found so far show a dominant effect, but recessive mutations are likely to also contribute a sizeable fraction of cases [71,92]. In addition, there is likely to be an important role for modifying mutations in the genetic background that can alter the phenotypic expression of the 'primary' mutation. This is the norm, even for the most classically 'Mendelian' disorders [35,77,93]. One should thus expect a distribution of genetic mechanisms across cases - some will be caused by highly penetrant mutations, others by mutations with more variable outcome, which are modified to some extent by the genetic background, and yet others will involve the inheritance of two or more distinct mutations [35,77,94-96].

Sixth, the eventual phenotype will also be modified by non-genetic factors, including (i) intrinsic developmental variation, where the phenotypic outcome varies due to inherent noise in the molecular processes mediating neurodevelopment [97], and (ii) environmental risk factors, which have been implicated by epidemiological studies. For schizophrenia, for example, these include maternal infection, urbanicity, migration and cannabis use [98]. The impact of such factors in individuals may be highly uneven and dependent on genotype.

Despite these complexities, the major finding is clear: mutations with large effect on risk of psychiatric disorders exist and we now have the means to identify them. If we think of this as a genetic screen for mutations causing a specific phenotype, with demonstrated saturation mutagenesis of the human population [32], then the most effective approach to follow up these discoveries is clear: find the mutations of largest effect and use these as entry points to elucidate the underlying biology.

## *Bone fide *models of genetic etiology

Advances in genetic engineering allow us to recapitulate human mutations in mice, and increasingly in rat. It is possible to generate animals with full gene knock-outs, conditional removal of the gene with spatial and temporal control, knock-in of exact human alleles, and to precisely engineer genomic deletions or duplications into syntenic regions of mouse chromosomes, thereby yielding animal models with direct construct validity; that is, where the manipulation is an actual cause of the condition in humans [99,100]. An emerging principle is the importance of allelic specificity for phenotypic outcome. For example, mouse mutants of *Nlgn3 *and *Shank3 *that recapitulate human alleles found in autism patients demonstrate selective effects that differ from null alleles [101-103]. Mice modeling human deletions or duplications can also be generated [104-106], and phenotypically compared with mutations of single genes in the affected regions in order to track down the specific culprits [107,108].

The number of models generated to date that directly recapitulate or mimic the effects of human alleles is small, but they have already begun to reveal details of the mechanisms of pathogenesis in specific mutants and to highlight convergent pathways, as well as some important general principles. For example, mutations in *Nlgn*, *Nrxn *(encoding a neurexin), *Shank3 *(encoding an adaptor protein that interacts with the cytoplasmic tails of Nlgn proteins) and *Cntnap2 *(which also encodes a member of the neurexin protein family) all affect synapse formation, altering the biochemical composition of synapses and the balance between excitation and inhibition between specific cell types in developing neuronal networks, with a range of concomitant behavioral deficits [101-103,109-112]. Local and long-range circuitry can also be altered by defects in cell migration and axon projection, which have been observed in *DISC1 *mutants or transgenic lines (for example, [113,114]), paralleling observations in humans with *DISC1 *mutations [115]. Alterations in long-range functional connectivity have also been observed - for example, between cortex and striatum in *Shank3 *mutant mice [111], between cortex and hippocampus in *Df(16)A^+/- ^*mice, which recapitulate 22q11.2 deletion [105], and in cortical synchrony in *Cntnap2 *mutant mice [112]. These kinds of observations help generate specific hypotheses regarding the cellular origins of pathophysiological states, which can now be tested with the increasingly sophisticated neurogenetic tools available (for example, [116-118]).

Studies in some animal models, such as mice mutant in *Fmr1 *or *MeCP2 *(the gene for methyl CpG binding protein 2, mutated in Rett syndrome), have even suggested possible new therapies based on detailed understanding of the biochemical pathology [119-121]. Despite all these advances, however, the complete pathways of pathogenesis for any single case remain obscure. Even for *Fmr1*, where the effects of mutation of this gene on synaptic plasticity are well described at the biochemical and cellular levels, the ultimate impact of these changes on the functions of specific neural circuits and how these explain the observed behaviors or psychopathology are only beginning to be unraveled [122].

To fully elucidate the pathogenic mechanisms for any specific mutation will require the integration of analyses across multiple levels, from protein function to systems neuroscience. What are the biochemical functions of the mutated protein? What are the cellular effects in various brain regions? How do these affect the function of various neural circuits? How do changes in neural activity play out over subsequent activity-dependent development? What are the cascading or reactive consequences as the brain develops with these initial alterations? What are the concomitant effects on physiology and behavior? Which circuit defects are responsible for which behavioral phenotypes? Which are the ones most relevant to the symptoms of the human disease? Which are the ones most amenable to intervention? These are formidable challenges even in light of the spectacular advances in modern neuroscience.

Rather than predefining which areas or mechanisms are important or how we expect animal models of psychiatric disorders to behave, we can take a bottom-up approach and simply ask, for any of these mouse mutants 'what happens to their brain circuits?' In analyzing the effects of any particular mutation it will be essential to consider the full cellular complexity of the circuitry and to use all the tools of modern molecular neuroscience to dissect the causal chains from primary genetic lesion to ultimate phenotype(s).

## A cell type-based approach is key to link genes and circuits in discovering pathogenic mechanisms

**Figure 1:**
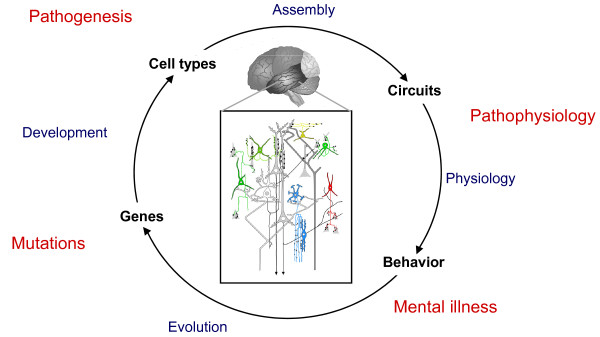
**A framework to elucidate pathogenic mechanisms from mutations to mental illness**. The effects of mutations in different genes can be analyzed across development on diverse cell types, local circuit organization and emergent properties, functional connectivity on a macro scale and correlated behavior in animals or psychopathology in humans.

Is there a generally useful strategy to tackle the problem of circuit pathogenesis in rodent models? If genes are the entry points to the cause and genetic architecture of psychiatric disorder, then we suggest that specific cell types may be the next set of entry points in neurobiology studies that may coherently link different levels of analysis, integrate different approaches and technologies, and yield useful answers in understanding pathogenic mechanisms. This is based on the following three first principles: individual cell types are the basic components of neural circuit organization, the building blocks of circuit assembly, and the basic units of gene expression and regulation in the brain (Figure 1). Therefore, a research program centered around cell type-based approaches is likely to be most productive in discovering the biological processes that build and operate neural circuits, and in revealing how these processes are altered by mutations and environmental insults.

The focus on cell types embraces the importance of different types of synapse. For example, mutations in *Nlgn *genes differentially affect the activity-dependent stabilization of excitatory (*Nlgn1*) or inhibitory (*Nlgn2*) synapses, selectively altering levels of different neurotransmitter receptors [123-125]. The effect of *Nlgn2 *mutation is specific for inhibitory synapses from fast-spiking (parvalbumin-positive) but not from somatostatin-positive interneurons [126]. Such effects can also be region-specific: *Nlgn3 *knock-in mice show increased synaptic inhibition in the cortex, but increased excitatory synaptic transmission in the hippocampus [102]. Mutations in other genes, such as *Fmr1 *[122] and *ErbB4 *or *Nrg1 *[127], show similarly cell type- and synapse type-specific effects. Understanding the particular profile of effects across multiple cell types in each mutant thus requires engagement with the cellular complexity of these circuits. This profile will also importantly include compensatory changes that may be secondarily induced and that could play important roles in pathogenesis (for example, [128-131]).

However, cell type-based analysis of neural circuits, although more routinely practiced in invertebrate systems, is highly challenging in the mammalian brain circuits, which consist of highly diverse and intermingled cell types that, until recently, could not be readily identified and experimentally accessed.

Fortunately, our ability to carry out such cell type-based analyses is rapidly increasing, as new neurogenetic tools are developed. Gene expression profiles that define many cell types have been elucidated, enabling researchers to isolate specific genetic elements that can be used to drive cell type-specific expression of a variety of transgenic constructs with different uses. These include tracing axonal projections and synaptic connections, monitoring electrical activity, and non-invasively activating or shutting off neurons in behaving animals with optical methods or chemical ligands [117,132,133]. Combined with advances in multi-photon imaging, automated microscopy and computational resources to deal with very large datasets, these tools confer unprecedented power to dissect the structure and function of neuronal circuits [134,135].

The Cre-loxP system has been adopted as a standardized approach in the mouse to drive such constructs in specific cell types [136]. This binary gene expression system is an effective strategy that confers cell type specificity as well as combinatorial power. A Cre driver mouse line provides a 'genetic switch' in a cell type that, through site-specific recombination between loxP recognition sites, can turn on or off the various molecular probes that allow these cells to be analyzed by all the above modern techniques in their native circuit *in vivo*. An increasing number of Cre drivers thus will allow more systematic and integrated analysis of different cell types in the relevant circuits (for example, [137,138]). Furthermore, certain Cre drivers allow tracking the developmental history of cell types, thereby enabling a comprehensive analysis of these cell types, from their specification, migration and synapse development to their integration and function in the circuits. This approach will be particularly powerful in revealing how the developmental trajectories of cells and circuits are altered, what are the primary cell autonomous changes due to 'direct hit' of the mutant gene, and what are the secondary, possibly maladaptive changes that lead to aberrant circuit-level operations. Genetic tracking of the development of distinct cell types thus will begin to link studies of circuit assembly and function and their alterations in disease models. This can be achieved simply by bringing the Cre driver allele and relevant reporter alleles into an etiology model through breeding.

Analysis of multiple circuits brain-wide in a disease model will allow a more unbiased 'screen' for deficits, less restricted by prior knowledge and assumptions of disease pathology. This will allow definition of the overall profile of deficits across multiple systems in each individual mutant, which may be quite unique. Comparison of multiple disease models promises to identify shared or distinct circuit deficits in these models, which may underlie their shared or distinct behavioral phenotypes. To achieve this, it will be increasingly useful to establish high-throughput methods to effectively screen for alterations in cell numbers, connectivity, and function with cell type resolution [134].

The convergence of phenotype across multiple animal models may arise at the biochemical or cellular level in some cases (such as pathways involved in synapse formation [87,88] or in cAMP signaling [139]), but only at the level of the emergent functions of large-scale neuronal networks in others (such as the responsiveness of the dopaminergic system [129,140] or GABAergic signaling and neuronal synchrony in the prefrontal cortex [131]). A central challenge in this framework is thus to link the cellular level analysis of phenotypic effects on microcircuit architecture to the functional consequences for brain function on a larger scale. It is these emergent functions that will best explain behavior or psychopathology. Rather than merely demonstrating that a mouse mutant shows some patterns of behavior reminiscent of human symptomatology, it will be essential to analyze physiological measures of circuit dynamics that explain behavior on a moment-to-moment basis in the relevant social and cognitive context.

## Large-scale analysis of circuits that subserve behavior

The neuroscience of animal and human behavior has witnessed a productive paradigm shift in the past decade where the trend of assigning complex traits to single genes has given way to the realization that dynamic coordination between micro and macro circuits is key to understanding the neuronal basis of behavior [141]. In other words, the emphasis has shifted from associating a behavior to a single gene or its protein product simply because mutation of that gene disrupts that behavior to understanding how functional variability at the gene or protein level influences the dynamic coordination of neurons in systems that support a certain behavior.

In the context of schizophrenia, the approach has been descriptive and mechanistically naive in that, for the most part, it has focused on associating a common allelic variation, which in some cases has not even been associated with a functional alteration, to complex behavioral disruptions whose physiology is still not fully understood. It is now increasingly being appreciated that a mechanistic understanding of the pathophysiology of schizophrenia is contingent upon (i) a better understanding of the dynamic coordination of neuronal processes that serve the behaviors that are disturbed in at least some cases of schizophrenia and (ii) understanding the functional role of implicated genes in micro and macro neuronal circuits that subserve this physiology. Recent studies, highlighted below, are beginning to make considerable advances in these two approaches.

Electrophysiological recordings using electroencephalography (EEG) have for decades reported behaviorally relevant oscillatory activity throughout the cerebral cortex [142]. Until recently, however, these data did not receive much attention in the context of psychiatric disorders, mostly because these measures had poor spatial and temporal resolution and were therefore considered functionally vague. The advent of magnetoencephalography (MEG) and development of better EEG instrumentation combined with invasive electrophysiological recordings in laboratory animals has now provided strong evidence that these measures are biologically meaningful correlates of behavior [143]. This has been in part because of a series of elegant studies in animals establishing a relationship between single neuron activity and local field potential oscillations in behaviorally relevant contexts [144,145].

Animal studies have further demonstrated that synchronized neuronal oscillations at various frequencies are a measure of coordinated neuronal activity that supports behavior. EEG and MEG studies in humans have subsequently established a close relationship between oscillations at various frequencies and behavioral performance, including working memory and selective attention [146]. This has been followed by several studies reporting changes in neuronal oscillations associated with cognitive deficits as well as some symptoms of schizophrenia [147-150]. EEG and MEG are thus promising translational methods that assess oscillatory activity with similar temporal and spatial resolution to more invasive methods that can be used in behaving animals, as described later.

Thus, experimental procedures such as pharmacological and genetic manipulations can be applied in conjunction with single unit recording and local field potentials to understand the molecular and cellular processes that could contribute to abnormal oscillatory activities [151,152]. The emergent properties of neuronal networks depend crucially on the cellular architecture of the microcircuits involved and on the interactions between definable cell types. For example, the connectivity between parvalbumin-positive interneurons and pyramidal neurons is essential to drive oscillations in the gamma frequency, for phase-locking between rhythms at different frequencies and for long-range temporal coherence between brain areas, which subserves cognitive and perceptual functions [116,151,153-157]. Other interneuron subtypes have dissociable functions on neural systems [158-161].

The proliferation of cell type-specific genetic drivers and development of optogenetic approaches also enable follow-up experiments to directly test hypotheses arising from studies of mutant phenotypes by manipulating the activity of the implicated cell types in highly specific ways [117]. Such approaches should allow the field to move beyond correlations between various anatomical and physiological disturbances in mutant animals to direct tests of how the precise connectivity patterns and strengths between different cell types affect the information-processing parameters of microcircuits and larger neuronal networks and impact on specific behaviors [116,118,154,162].

While mice are likely to continue to lead the way in the discovery of the effects of specific mutations on nervous system development and function, we can also expect the increasing use of genetically manipulated rat and primate models to further dissect pathophysiological mechanisms. Zinc-finger nucleases can be used to specifically modify the genome in effectively any type of cell, including embryonic stem cells or early embryos, thus generating genetically modified organisms [163]. This approach has been used to generate gene knockouts [164] and also to knock in specific alleles in the rat [165,166], opening up the possibility of recapitulating human alleles in this species and others in the future. Brain cells can also be genetically modified in a cell type-specific manner by viral transfection [132,167], making them accessible to acute gene manipulations and also to optogenetic techniques in any species, including rats and non-human primates [168,169]. These species, which have a much richer tradition of systems neuroscience and greater cognitive sophistication than the mouse, will no doubt be essential in further elucidating general principles and mechanisms of pathophysiology and relating them to humans.

## Translation to humans

Because diagnostic classification of mental illnesses in clinical psychiatry at present is merely based on phenomenology and not disease etiology, it is important to consider translation between humans and rodent models not via clinical diagnosis but specific biological traits. These traits would include behavioral constructs and physiological characteristics, and are crucial in understanding disease mechanisms in rodent models. Even more importantly, such characteristics can be used as indicators of drug screening in rodents and markers in clinical trials with humans.

A major confound in analyzing neurobiological intermediate phenotypes in psychiatric patients has been the extreme underlying (and previously cryptic) genetic heterogeneity. As the genetic factors involved in psychiatric illness are determined in more and more patients, it should be possible to make much more direct and powerful comparisons of underlying phenotypic traits between genetically defined subsets of humans and mice carrying the same mutation. This genetic stratification of patients may also prove invaluable in the design of clinical trials aimed at primary causes. On the other hand, the analysis of phenotypes correlated with clinical states will need to look for convergence across mouse mutants or human patients with diverse genetic causes.

Many efforts have been made to try to identify equivalent characteristics between humans and rodents. For example, at the behavioral level, the Cognitive Neuroscience Treatment Research to Improve Cognition in Schizophrenia (CNTRICS) aims to develop and implement animal model paradigms that can tap the cognitive and emotional-processing constructs translatable to humans [170]. Although only limited numbers of paradigms are widely accepted to be translational, collaboration among human neuropsychologists and animal neurobehavioral scientists may identify several paradigms to be utilized for translation. Starting these studies with animal models with direct etiological validity should tell us which behavioral constructs are really most relevant to human psychopathology, even if the effects manifest differently on the surface.

Given well-founded concerns over the species-specificity of behavioral measures, it seems likely that neurophysiological measures may prove to be more translatable between animals and humans. This may be especially true of measures of functional connectivity or coherence between brain areas, as these may most powerfully index differences in network function that affect psychopathology. These can be assayed with EEG and MEG, as described above, but also with functional connectivity studies using functional magnetic resonance imaging to examine correlations between activities of remote but connected regions [171-174]. Although these measures are simply about observed correlations, they provide clinically relevant dynamic information that can be invaluable for animal studies, which can in turn provide more detailed mechanistic information about the neuronal basis of the reported aberrant connectivity. For example, the reduced synchrony between hippocampal and prefrontal regions in the 22q11.2 deletion *Df(16)A^+/- ^*mice [105] parallels observations in humans with schizophrenia [171], generating testable hypotheses about the origins of this defect [157]. Alterations in cortico-striatal or intracortical coherence in *Shank3 *[111], *Fmr1 *[122] and *Cntnap2 *mutant mice [112] similarly provide entry points to define the pathophysiology of these disorders at a level that can be directly assessed in humans, especially those with mutations in the same genes.

Definition of the mouse correlates of human pathophysiology and psychopathology will also allow a reverse translational approach to identify new candidate susceptibility genes. Prospective phenotyping of mice with mutations in neurodevelopmental genes may reveal phenotypes paralleling established models (for example, *NogoA *[175], *Dlg4 *[176], *Sema6A *[177] and *Slitrk5 *[178]). This gives a higher prior probability for assigning pathogenicity in the event that mutations in those genes are then found in human patients.

## Strategies towards new treatments

The drugs currently used to treat disorders like schizophrenia were discovered serendipitously, are only partially effective for positive symptoms but not the more debilitating negative and cognitive symptoms, work in some patients and not others and have serious side effects [179]. For autism spectrum disorder, the situation is even more bleak - while there are some drugs prescribed to treat some symptoms there is no overall therapeutic of proven value [180]. The main reason hardly any new drugs (with novel mechanisms) have been successfully developed in the past 60 years has been our lack of understanding of the underlying mechanisms of these disorders.

New genetic models may be utilized in identifying novel treatment strategies in three ways: first, to identify novel targets through which we can find more effective and safer compounds for treatment within the current understanding of these diseases; second, to identify novel targets and compounds that may not be obvious from current paradigms; third, and probably most important, to identify targets and compounds for early intervention.

By analyzing molecular, cellular and circuitry disturbances in new genetic models, we may find better clues to how the cellular pathways involving the gene of interest may interact with currently accepted pathophysiological paradigms (such as the roles of GABA, NMDA receptors and dopamine receptors in schizophrenia). In such cases, we may identify better drug targets, against which we can expect compounds with better efficacy and less side effects.

However, our hope is to identify new targets and compounds with new genetic models. Although investigators who generate new genetic models tend to test whether current medications (such as clozapine and haloperidol for schizophrenia) can normalize deficits in behavioral paradigms, it may be more advantageous for new drug discovery to identify paradigms that are relevant to human diseases (that is, likely to correlate not to one disease but to several), but that cannot be normalized by current medications. For example, a *DISC1 *transgenic mouse model with neonatal poly(I:C) treatment (to mimic maternal infection) shows many types of behavioral deficit, most of which are normalized by administration of clozapine [181]. However, the impairment of social behaviors in this model is resistant to both clozapine and haloperidol treatment. Provided that these impairments are, at least in part, relevant to negative symptoms, screening of novel compounds to normalize this behavior in this model may lead to the identification of new treatments for negative symptoms.

Early intervention is now becoming a key in treatment strategies in many brain disorders, such as Alzheimer's disease and schizophrenia. Animal models, especially those in which neurodevelopmentally important genes are targeted, are very useful to dissect the pathological course even from the premorbid stage to the full onset. For some symptoms, specific common phenotypes might be due to secondary mechanisms by which the developing brain reacts to diverse primary insults. For example, altered dopaminergic signaling may arise as a result of a variety of primary defects and may represent a final common pathway to psychosis [129,130,140]. The molecular homeostatic mechanisms underlying these secondary changes may thus represent a viable target for early intervention in genetically at-risk subjects. Similar events may underlie the emergence of dysfunction in other circuits across other disorders. In the case of schizophrenia where the intervention has to occur during early to late adolescence, the major obstacle to moving intervention to an earlier age is concerns over the effects of manipulating brain systems that are still developing. These concerns are compounded by a lack of concrete understanding of the biology of adolescent brain, especially in the context of the physiology of affect and cognition. Animal models will, therefore, be critical in advancing this aspect of the field.

## A way forward

The discovery of mutations that strongly predispose individuals to psychopathology provides a crucial starting point to define pathogenic mechanisms and pathways. Direct animal models of genetic etiology can be analyzed in a comprehensive and systematic way, using the full arsenal of modern neuroscience to link effects on particular cell types in defined microcircuits to emergent properties of larger scale brain networks in behaviorally relevant contexts. These analyses will hopefully reveal neurobiological phenotypes that can be translated very directly to humans, suggest points of possible therapeutic intervention and aid the rational design of new drugs targeted at the underlying causes of mental illness.
